# Vitamin Derived Nitrogen Doped Carbon Nanotubes for Efficient Oxygen Reduction Reaction and Arsenic Removal from Contaminated Water

**DOI:** 10.3390/ma13071686

**Published:** 2020-04-04

**Authors:** Vadahanambi Sridhar, Kwang Hyo Jung, Hyun Park

**Affiliations:** 1 Global Core Research Centre for Ships and Offshore Plants (GCRC-SOP), Pusan National University, Busan 46241, Korea; sridhar@pusan.ac.kr; 2 Department of Naval Architecture and Ocean Engineering, Pusan National University, Busan 46241, Korea; kjung@pusan.ac.kr

**Keywords:** Fe-N-C, ORR, microwave synthesis, vitamin, arsenic removal

## Abstract

Nitrogen doped carbon nanotubes (NCNT) that were prepared by simple microwave pyrolysis of Niacin (Vitamin B_3_) as noble metal free electrocatalyst for oxygen reduction reaction (ORR) is reported. Our newly developed technique has the distinct features of sustainable and widely available niacin as a bi-functional source of both carbon and nitrogen, whereas the iron catalyst is cheap and the fourth most common element in the Earth’s crust. The results of the electrochemical tests show that our newly developed iron impregnated NCNT anchored on reduced graphene substrate (Fe@NCNT-rGO) catalyst exhibit: a positive half-wave potential (E_1/2_) of 0.75 V vs. RHE (reversible hydrogen electrode), four-electron pathway, and better methanol tolerance when compared to commercial 20% Pt/C. When applied as adsorbent for arsenic removal, our newly discovered NCNT-Fe illustrate the efficient and effective removal of arsenic across a wide range of pH values.

## 1. Introduction

With fast depletion of fossil fuels, there is an immediate need for clean, green, and energy efficient fuels. Fuel cells, which are capable of high energy densities, have recently emerged as environment-friendly power generation devices. However, the problem of reliable, low-cost and efficient catalysts for the oxygen reduction reaction (ORR), is still a major hurdle thereby limiting their application. Till date, platinum nanoparticles dispersed in carbon matrix, colloquially known as Pt/C electrodes, show good performance in ORR reactions, but suffer from disadvantages, like high prices and poor cycling ability attributed to the deactivation of catalyst due to the formation of metal carbonyls. Therefore, there is a need for alternatives that are based on non-noble metals. Amongst the various alternatives, elements of iron triad, namely iron [1,2], cobalt [3,4] and nickel [5,6], and ntrogen-doped carbon materials [7,8], have been extensively explored as low-cost and high-performance electrocatalysts. Amongst these, iron nano-particles that are embedded in nitrogen doped conductive carbon nanostructures have aroused tremendous interest due to its excellent catalytic activity. These kinds of non-precious metal/nitrogen doped carbon nano-material systems are fast emerging as the most probable replacement for the expensive and inefficient commercial Pt/C ORR catalysts [9,10]. Theoretical calculations [11] and experimental studies have both shown that the presence of metal–nitrogen (M–N_x_) complexes embedded in conductive carbon skeleton is critical factor in obtaining high electrocatalytic activity in ORR. Amongst the various metals investigated, there is growing consensus that highly dispersed iron-nitrogen complexes embedded in carbon nanomaterials are good catalysts for ORR reactions in both acidic and alkaline electrolytes [12,13]. Besides these, metal free nitrogen doped CNT synthesized from the pyrolysis of iron and cobalt phthalocyanine compounds [14] have also been reported.

Besides the catalyst morphology, the substrate on which the catalyst particles are dispersed is another crucial factor that determines the number of accessible active sites and the degree of mass transfer in the ORR [15,16]. For this, a wide variety of carbon material substrates with various morphologies, like graphene [17], carbon nano-coils [18], carbon nanotubes [19], and carbon nanosheets [20], have been widely explored as hosts for Fe–Nx catalytically active sites. Among these, two-dimensional (2D) carbons, especially graphene and its analogues, like carbon nanosheets, which exhibit attractive structural advantages, such as high surface area, excellent electrical conductivity, and superior mechanical/chemical stability, have been studied [21]. However, the tendency of 2D graphene to restacking due to van der Waal’s forces especially in polar solvents like water and alcohols generally eclipses the desirable properties like high surface area and porosity. Therefore, it is advantageous to have a catalyst with a three-dimensional, meso-porous structure that exhibits good surface area, thereby exposing active catalytic sites to an electrolyte. For this, three-dimensional conductive graphene-carbon nanotubes mesoporous structures would be an ideal choice. Amongst the various methods for synthesis of nanomaterials, microwave synthesis is rapidly evolving as a reliable and convenient method that requires special mention [22,23,24]. With respect to microwave synthesis of carbon nanotubes, we have reported the formation of nitrogen doped carbon nanotubes from various precursors, like azobis compounds [25], imidazole [26], and Zeolitic imidazolate frameworks [27].

The pollution of groundwater with arsenic being included by World Health Organization (WHO) as one of the 10 chemicals of major public health concern is a worldwide problem. High levels of arsenic exceeding the WHO’s recommended limit of 10 µg/L has been observed in ground waters of Argentina and Chile in South America, South West of USA (Arizona, California, Colorado, Nevada, New Mexico, and Utah), Northern and central regions of Mexico, Hungary and Romania in Europe, Xinjiang, Inner Mongolia, Henan, Shandong, and Jiangsu provinces of China, Ganga-Brahmaputra river basin of India and Bangladesh, Mekong delta region of Vietnam and Cambodia, Southern regions of Thailand, Rift valley of Ethiopia and Eritrea, almost all regions of South Africa, Botswana, and Zimbabwe, Niger river basin of Nigeria, Togo, Ghana, and Benin, thereby exposing millions of people to various cancerous (bladder and lung cancer) and non-cancerous health effects, such as high blood pressure, diabetes, and skin lesions, etc. Scientists and technologists have devised various strategies to effectively eliminate arsenic from groundwater in order to combat this global menace, which can be broadly divided into adsorption, coagulation-precipitation, filtration, flocculation, ion-exchange and reverse-osmosis techniques. Even though arsenic removal can also be integral part of traditional water treatment techniques like coagulation-precipitation, filtration, reverse osmosis (RO), etc., but these techniques are only effective in scenarios wherein the concentration of arsenic is higher than 100 µg/L and at lower concentrations, the efficiency of aforementioned techniques for the removal of arsenic is less than satisfactory. Additionally, the cost that is associated with these processes is also pretty high. According to a report by USEPA (United States Environmental Protection Agency), in towns with more than 10,000 people, the operating cost of ‘centralized arsenic removal’ facility will range from $0.86 to $32 per household and for small villages with 25–500 inhabitants, the cost will skyrocket from $165 to $327 per household [28]. Therefore, there is a need for economical and effective removal of arsenic. In this manuscript, we investigate the utility of our newly developed iron embedded in nitrogen doped carbon nanotubes anchored on carbon fiber (Fe@NCNT-CF) as cost-effective adsorbent for removal of arsenic from contaminated water.

Herein, we report synthesis of iron nitride/iron carbide nano-particles embedded in mesoporous three-dimensional (3D) nitrogen doped carbon nanotubes that were anchored on reduced graphene substrate (Fe@NCNT-rGO) by a simple microwave radiation using niacin, vitamin B_3_ as the sustainable source of nitrogen doped carbon. We have reported the microwave synthesis of nitrogen doped carbon nanotubes from synthetic precursors, like ACHN (azobis(cyclohexanecarbonitrile)) [25], imidazole [26], and its derivatives, like 2-methylimidazole based zeolitic imidazolate frameworks (ZIF) [27], but the utility of nitrogen doped sustainable resources has been less investigated. Here, in this work, we show that niacin, vitamin B_3_ can be a good source for nitrogen doped carbon nanotubes. When applied as electrodes in ORR reaction, our results show that Fe@NCNT-rGO deliver superior activity to ORR when compared to that of the commercial Pt/C with almost the same onset potential and a ~10 mV higher half-wave potential (ΔE_1/2_). The utility of our newly discovered niacin precursor for synthesis of NCNT with other catalysts, like cobalt, and on microwave susceptible surfaces, like carbon fibers and its utility for removal of arsenic from contaminated water are also demonstrated. Our newly developed technique is not only fast, but it can also be scaled up to the gram scale production of functionalized carbon nano-hybrids for energy and environmental applications.

## 2. Experimental

### 2.1. Materials and Methods

99.5% pure graphite (Item number: ES 350 F5) was purchased from Samjung (C & G, Gyeongsan, Korea), whereas reagent grade sulphuric acid (H_2_SO_4_), hydrochloric acid (HCl), sodium nitrate (NaNO_3_), hydrogen peroxide, potassium permanganate (KMnO_4_), Niacin and iron(III) acetate, and cobalt acetate were purchased from Sigma-Aldrich, Seoul, Korea), and they were used as received. Microwave irradiation was carried out in a domestic microwave oven that was manufactured by Daewoo, Seoul, Korea, Model number: KR-B202WL with output power of 700 W operating at 2450 MHz. The morphology of the synthesized catalysts were tested with a field-emitting scanning electron microscope (SEM) (Zeiss FEG-SEM Supra 25, Seoul, Korea), whereas high resolution transmission electron microscopic (TEM) images, High-angle annular dark-field (HAADF), and elemental Energy Dispersive Spectroscopy (EDS) maps were recorded on TALOS F200X (Thermo Fisher Scientific Korea Ltd., Seoul, Korea). The operating voltage was 10 and 200 kV, respectively, in SEM and TEM. Surface area and porosity measuements were carried on an Belsorp Mini II Surface Area analyzer (Microtrac MRB, York, PA, USA) at –196 °C. Structural analysis by Raman spectra and X-ray diffraction (XRD) patterns were recorded on LabRAM HR evolution confocal Raman spectrometer (Horiba France SAS, Longjumeau, France and Rigaku D-max diffractometer (Rigaku, Tokyo, Japan), respectively. The electronic states of chemical moieties were studied by a Sigma Probe Thermo VG X-ray photoelectron spectrometer (Thermo Fisher Scientific Korea Ltd., Seoul, Korea) and XPSPEAK ver 4.1 (downloaded from: https://xpspeak.software.informer.com/4.1/) was used for curve fitting. Prior to testing, the sample was first degassed for 24 h at 250 °C.

All of the electrochemical measurements were conducted on a CHI 660 electrochemical station (CH Instruments, Inc. Austin, TX, USA) with a conventional three-electrode system. In a typical electrochemical measurement, a total of 5 mg of catalysts was dispersed by sonication in a mixture of 480 μL isopropanol (J&K Scientific LTD, Ansan, Korea) and 20 μL of Nafion aqueous solution (5 wt.%, DuPont Korea Inc., Seoul, Korea for 30 min. 12 μL of the above suspension was gently dropped onto a glassy carbon rotating disk electrode (RDE, 5 mm diameter) or rotating ring-disk electrode (RRDE, 4.93 mm inner diameter and 5.38 mm outer diameter) and then dried naturally in the air. The above procedure was repeated twice, which results in a total loading mass of approximately 0.6 mg/cm^2^. The electrolyte was 0.1 M KOH aqueous solution, and the reference and counter electrodes were saturated calomel electrode (SCE) and Pt wire, respectively. The electrolyte was saturated with O_2_ before the experiment, and O_2_ was continuously supplied during the experimental operation. All of the potential values reported in this study were converted to the reversible hydrogen electrode (RHE) scale, according to the equation: E_RHE_ = E_SCE_ + 0.0591pH + 0.242. For the stability test, the working electrode ran at −0.7 V vs SCE for 60,000 s in O_2_-saturated 0.1 M KOH with a rotation rate of 1600 rpm. For comparison, commercial Pt/C (20 wt.% Pt) powder was tested under the same conditions, with a loading mass of 0.25 mg cm^−2^. Linear sweep voltammetry (LSV) was measured by the RDE/RRDE technique the with the scan rate of 10 mV/s at various rotating speeds, from 400 to 2400 rpm.

The As(III) solutions were prepared by dissolving sodium arsenite (NaAsO_2_) in double distilled water and subsequently deoxygenated by passing 99 % pure for 15 min. and the adsorbent Fe@NCNT@CF was added immediately. All of the adsorption experiments were carried out in closed, 120 mL bottles maintained at 25 °C, and the pH of the solution was varied by adding NaOH and HCl.

### 2.2. Synthesis of Nitrogen Doped Carbon Nanotubes from Niacin

The synthesis of 3D carbon nano tubes that were anchored on graphene was carried out in a ‘one-pot’ microwave technique. Briefly, 10 g of niacin and 2 g of iron(III) acetate were added to a 2 g of graphene oxide (synthesized by a modified Hummer’s method [29]) that was dispersed in 500 mL of ethanol. This solution was refluxed at 50 °C for 300 min. and subsequently dried in an oven to remove ethanol to obtain a viscous semi-solid that was placed in a glass vial and subjected to microwave irradiation at 700 W for 200 s to obtain a fluffy powder. The obtained product was washed with ethanol and de-ionized (DI) water to remove any traces of any unreacted niacin and sufficiently dried in an oven to obtain Fe@NCNT-rGO.

## 3. Results and Discussion

**Figure 1:**
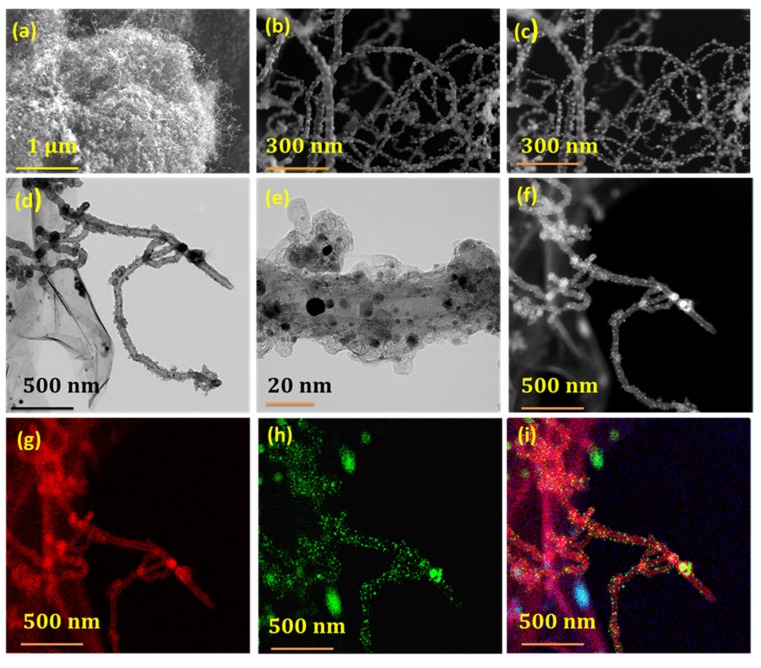
SEM micrographs at low and high magnification (**a**,**b**) and corresponding secondary electron image ((**c**)) of Fe@NCNT-rGO. TEM image at low (**d**) and high (**e**) magnification exhibiting predominant presence of iron nano-particles anchored on CNT; (**f**) shows high-angle annular dark-field (HAADF) image and its corresponding nitrogen (**g**), iron (**h**) and combined map (**i**).

The microstructure and morphology of Fe@NCNT-rGO was studied by SEM at small magnification (Figure 1a), which show evenly distributed and high density growth of carbon nanotubes on the graphene substrate. Additionally an extensive presence of iron nano-particles encapsulated on the surface of CNTs can also be observed in high magnification SEM micrograph exhibited in Figure 1b wherein the iron nano-particles appears brighter than the surrounding carbon nanotubes, which become further evident from corresponding ‘secondary electron’ image exhibited in Figure 1c. Back-scattered electron (BSE) images are beneficial in the study of multi-component nanostructures different chemical compositions when compared to the conventional ‘through-the lens’ or ‘immersion-lens’ images. Iron nano particles backscatter electrons more strongly due to their higher atomic number when compared to the underlying carbonaceous matrix, thereby appearing brighter in the recorded image. The bright iron nanoparticles are well dispersed and distributed along the walls of carbon nanotubes, whereas the larger particles that were enclosed inside CNTs appear more brightly than their smaller counterparts.

A two-step procedure can be contemplated for the growth of carbon nanotubes from Vitamin B_3_. In the first step, the iron nano-particles react with oxygen containing moieties, like carbonyl, carboxyl, and hydroxyl groups of graphene oxide (GO), and reduce it to reduced graphene oxide (rGO). Subsequently, these Fe nano-particles anchor onto the intrinsic point and line defects generated chemically during the oxidation reaction and/or mechanical defects that are generated due to the prolonged sonication during the process of exfoliation. Secondly, due to the intense heat produced by localized microwave heating, niacin thermally decomposes to ringed aromatic and alkyl-aromatic hydrocarbons and linear nitrogen containing phenols, which, upon capture by the iron nano-particles, are dehydrogenized to form nitrogen doped carbon nanotubes by the dissolution-extrusion mechanism [27]. Furthermore the carbon source niacin is rich in nitrogen (about 8.9 molar wt%), the formed graphitic layers around the catalytic iron nanoparticles are tightly bound due to the high binding energy of nitrogen to iron, which is higher than the binding strength of carbon to iron, thereby forming defect rich, nitrogen doped carbon nanotubes (Figure 1d). The HRTEM image of a portion of CNT that is shown in Figure 1e reveals that the nanotubes are defect rich with irregular walls and the iron nano-particles are less than 20 nm in size and they are well distributed not only inside the nanotubes, but also on the walls of the CNTs. Besides, some iron nanoparticles are also enclosed in protective carbon enclosures, forming typical core-shell nano-structures. The yield of CNTs, as measured by weight gain method, was found to be 27.6 ± 0.5 wt%.

Figure 1f shows the corresponding HAADF image of Figure 1d, and the elemental nitrogen EDS map (Figure 1g) shows that the nitrogen moieties are well distributed along the walls of the nanotubes, whereas, from the EDS map of iron (Figure 1h), well dispersed and distributed nano-particles along the walls and also enclosed inside the nanotubes can be inferred. The composite map of nitrogen and iron shows that the majority of iron and nitrogen is in the carbon nanotubes when compared to the underlying graphene substrate.

**Figure 2:**
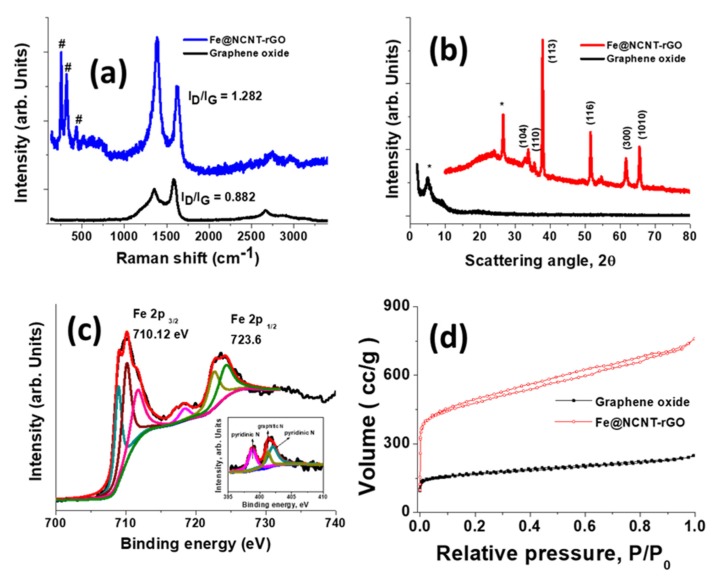
Raman spectra (**a**) and X-ray diffraction (XRD) (**b**) of graphene oxide and Fe@NCNT-rGO; Deconvoluted Fe 2p XPS spectra (**c**); BET surface area of graphene oxide and Fe@NCNT-rGO. Inset in (**d**) is deconvoluted N1 s spectra of Fe@NCNT-rGO.

The Raman spectra of graphene oxide and Fe@NCNT-rGO (Figure 2a) measured at 514 nm excitation frequency shows three peaks at 226, 496, and 538 cm^−1^ that correspond to iron moieties in Fe@NCNT-rGO. These peaks are in addition to the widely known graphene related peaks: the Raman active G band peak at 1584 cm^−1^, which is due to the in-plane vibrational mode and the disorder induced 1352 cm^−1^ peak due to the presence of carbon nanotubes on the graphene substrate. The 2D peak occurring in the vicinity of ~2600 cm^−1^ is sharper in Fe@NCNT-rGO and it is related to sp^2^-carbon bonds combined with a minor hump that is associated with disordered carbon occurring at ~2400 cm^−1^ [25]. Another interesting observation is the decrease in the I_D_/I_G_ ratio (ratio of intensities of D band to G band) from 0.882 in graphene oxide to 1.282 in Fe@NCNT-rGO, which proves that the defects generated on graphene oxide substrate during its synthesis are healed due to the growth of CNT. XRD was used to study the structure of graphene oxide and Fe@NCNT-rGO, wherein a single peak at the scattering angle of 2θ = 5.34 corresponding to d-spacing of 1.94 nm dominates the XRD plot of graphene oxides, whereas the diffraction pattern of Fe@NCNT-rGO is dominated by iron related peaks at 2θ values of 24.6 corresponding to crystalline carbon of CNT and at 33.7, 35.64, 37.95, 51.56, 61.62, and 65.5 corresponding to (104), (110), (1130, (116), (300), and (1010) of iron carbide and iron nitride moieties, which has been further confirmed from XPS studies [30].

XPS spectroscopy is an useful tool for studying the electronic and chemical structure of iron moieties and the deconvoluted XPS spectra of Fe 2p binding energy region is plotted in Figure 2c which shows two distinct sharp peaks at 710.12 and 723.6 eV corresponding to Fe 2p_3/2_ and Fe 2p_1/2_ electronic states. Of these two peaks, the Fe 2p_3/2_ is much narrower and stronger when compared to Fe 2p_1/2_. The Fe 2p_3/2_ peak can further be deconvoluted to three peaks centred at 708.9, 710.2, and 711.8 eV which correspond to iron–ligand covalency [31], Fe–N bond [32], and iron oxynitride moieties [33] respectively. The deconvolution of Fe 2p_1/2_ peaks shows two peaks at 722.7 and 724.4 eV, which correspond to iron carbide clusters [34] and Fe_3_C [35], respectively. Besides these, a minor shake up satellite at 718.46 eV can also be observed. XPS and XRD studies indicate that the iron moieties are predominantly either as iron nitride and iron carbides with minor iron oxynitride impurities. The deconvoluted N1s spectra shown as inset in Figure 2c exhibit two broad peaks that are centered at 398.8 and 401.4 eV, which correspond to pyridinic-N (N connected to two C) and graphitic-N (N connected to three C), whereas the minor peak at 401.2 eV can be attributed to pyrrolic-N (N part of a pentagon ring connected to two C). The nitrogen adsorption isotherms of the graphene oxide and Fe@NCNT-rGO plotted in Figure 2d exhibit the type I/II characteristics, especially in Fe@NCNT-rGO, which has marginally higher surface area of 758.88 m^2^ g^−1^ and a more pronounced hysteresis loop in the P/P_0_ range of ≈ 0.1–0.89, which indicates the presence of extensive micro/nano pores that are attributed to the ‘spacer’ functionality of CNT inhibits the restacking of reduced graphene oxide [26].

**Figure 3:**
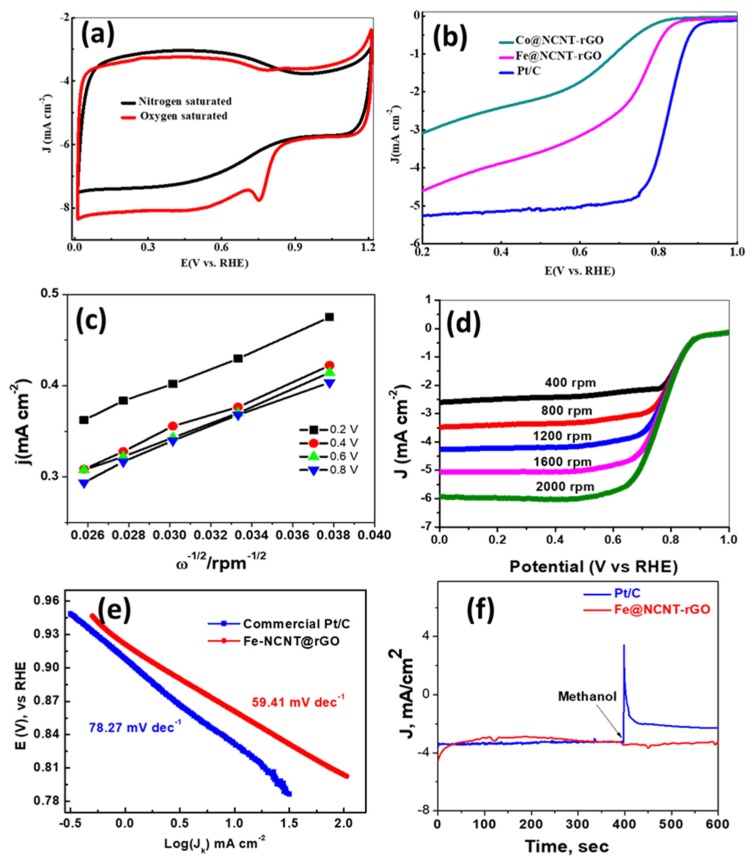
CV curves of Fe@NCNT-rGO electrodes in 0.1 M KOH saturated with N_2_ (black) and O_2_ (red) at a sweep rate of 50 mV s^−1^ (**a**); LSV curves of Fe@NCNT-rGO, Co@NCNT-rGO and commercial Pt/C in 0.1 M KOH at a rotation rate of 1200 rpm (**b**); corresponding K–L plots of Fe@NCNT-rGO at various potentials (**c**); LSV curves of Fe@NCNT-rGO measured at different rotation rates (**d**); Tafel plots (**e**); and, Methanol tolerance test (**f**) of commercial Pt/C and Fe@NCNT-rGO in 0.1 M KOH.

Figure 3a exhibits the electrochemical behavior of our newly developed vitamin B_3_ derived nitrogen doped CNTs on rGO were tested in nitrogen and oxygen saturated 0.1 M KOH electrolytes and the CV plot. Typical of Fe-N-C systems [36,37,38], the CV curve in oxygen saturated system shows a small hump at ~0.75V, whereas this hump is absent in nitrogen saturated electrolyte, which indicates the suitability of our Fe@NCNT-rGO for ORR type of reactions. A rotating disk electrode (RDE) was used to perform linear sweep voltammetry (LSV) in O_2_ saturated 0.1 M KOH electrolyte to measure the ORR activity and the kinetics of the Fe@NCNT-rGO, Co@NCNT-rGO, and commercially available Pt/C electrodes (Figure 3b). Amongst the three tested electrodes, Fe@NCNT-rGO exhibited outstanding electro-catalytic activity with a high onset potential of 0.96 V vs. RHE (reversible hydrogen electrode), which is close to that of commercial Pt/C catalyst. It can also be observed that both Fe@NCNT-rGO and Co@NCNT-rGO display slightly more positive half-wave potential (E_1/2_) of 0.87 V when compared to Pt/C (0.86 V). Even more, our Fe@NCNT-rGO exhibits slightly lower, but comparable, current density of 5.7 mA cm^−2^ when compared to the 5.9 mA cm^−2^ observed in commercial Pt/C electrodes. A diffusion controlled and effective four-electron ORR pathway can be inferred from the broad current plateau [39] that was observed in the region of 0.2 to 0.65 V. The RDE polarization curves were measured at increasing rotation speeds from 400 to 2000 rpm (plotted in Figure 3c) and the corresponding Koutecky–Levich (K–L) plots of the Fe@NCNT-rGO electrodes that are exhibited in Figure 3d show parallel linearity in the range of 0.2 to 0.65 V, validating the nearly first-order kinetics of the ORR reaction [40,41]. The number of electrons (n) transferred per oxygen molecule, as calculated by K-L equation, and was found to be around 4.0, which is close to the theoretical value of 4.0 for commercial Pt/C, which also reiterates our contention that four electron pathway is followed in our newly developed Fe@NCNT-rGO electrodes.

Figure 3e shows the Tafel slope value that is associated with the oxygen adsorption behaviour on the surface of the catalyst. The Tafel value for our Fe@NCNT-rGO was very close to that of Pt/C (78.27 mV dec^−1^ vs. 59.41 mV dec^−1^), suggesting a similar oxygen adsorption mechanism. The chemical composition of our Fe@NCNT-rGO, as measured from XPS survey spectra (Figure S1 of supplementary file), shows C: 85.33, N: 9.73, O: 1.79, and Fe: 3.12 wt%, which is dispersed and distributed as either Fe-N_x_ catalytically active nano-particles, and also as nitrogen moieties in bamboo shaped CNTs, enabling more effective and efficient catalytic activity and enhancing the mass transfer of ORR-related species.

The methanol tolerance test is also an important parameter for assessing the suitability of ORR electro-catalyst, especially for direct methanol fuel cells. This ‘methanol crossover’ generally reduces the ORR activity, which results in a lower efficiency of the fuel cell. The methanol resistance of our Fe@NCNT-rGO as compared to commercial Pt/C was evaluated in a chrono-amperometric test. Figure 3f shows the variation in relative current (I/I_0_) (ratio of the current measured at a given time to the initial current of the composite) as compared to that of commercial Pt/C. With the addition of methanol to the electrolyte solution at 400 s, a very sharp drop in the relative current was observed in commercial Pt/C, whereas in our Fe@NCNT-rGO, there was no substantial change in the relative current, which indicates excellent methanol tolerance. This can be attributed to the fact that most of the catalytically active Fe-Nx moieties in our Fe@NCNT-rGO are enclosed in a protective few graphene layers, as observed in high resolution TEM (HRTEM) (Figure 1e), thereby protecting them from oxidative damage. These characteristics, like high onset potential, smaller Tafel slope, and high methanol tolerance make our Fe@NCNT-rGO electrodes a better choice when compared to highly expensive commercial Pt/C electrodes.

**Figure 4:**
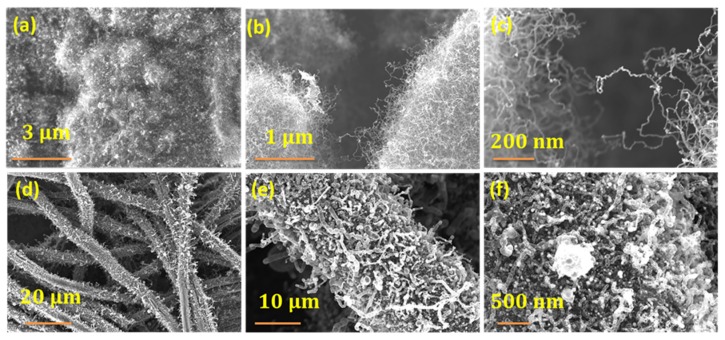
Representative SEM micrographs of Vitamin B_3_ derived nitrogen doped carbon nanotubes using cobalt catalyst (**a**–**c**) and CNTs grown on carbon fiber substrates (**d**–**f**).

Cobalt was investigated as a possible catalyst for microwave synthesis of NCNT from vitamin B_3_ in order to study the effect of catalyst. A similar synthesis procedure as described in Section 2.2 was employed with the only change being the replacement of iron catalyst with cobalt acetate. The choice of cobalt was governed by the fact that cobalt electrodes are also rapidly emerging as very effective ORR catalysts. Representative SEM micrographs at increasing magnification exhibited in Figure 4a–c show a similar morphology to that observed for iron catalyst (Figure 1). Commercially available carbon fiber (CF) was chosen in order to test the utility of our newly developed niacin to grow CNTs on other microwave susceptible substrates. The CFs were thoroughly washed with 10% HCl and DI water in succession and then dipped in iron-niacin stock solution for 30 min. in order to remove commercial ‘sizing’ coating. Subsequently, the fibers were removed and dried in oven at 70 °C to remove the solvent and then subjected to microwave irradiation for 60 s to yield nitrogen doped CNT decorated carbon fibers (Figure 4d–f)). Although CNTs grew on carbon fibers, the density was low due to the fact that intrinsic curvature of fiber surface is not conducive for the anchoring of catalyst particles. Besides, the nanotubes are thicker and the structure is more distorted when compared to that of graphene anchoring. The above two results exhibit the versatility of our newly discovered niacin as a novel precursor for the growth of carbon nanotubes on any microwave susceptible substrates.

**Figure 5:**
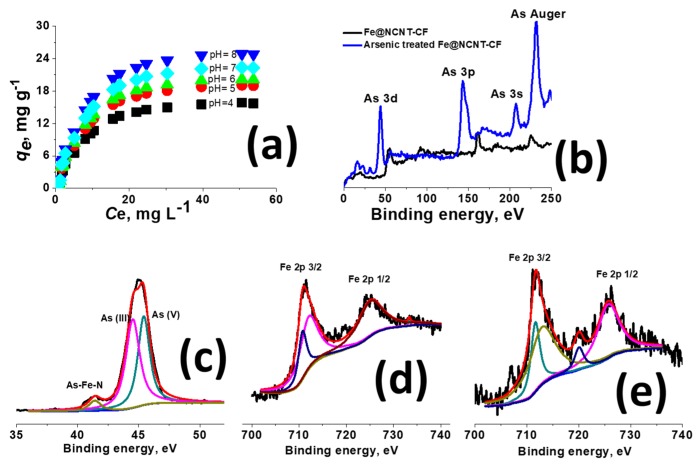
Adsorption isotherms of arsenic on Fe@NCNT-CF nanostructures at different pH values (**a**); Visible arsenic peaks at low binding energy of pristine and arsenic adsorbed Fe@NCNT-CF (**b**); deconvoluted spectra of As 3D (**c**); Deconvoluted Fe 2p spectra of Fe@NCNT-CF (**d**); and, arsenic adsorbed Fe@NCNT-CF (**e**).

The rate of adsorption of arsenic at pH values of 4 to 8, as plotted in Figure 5a, shows that, at all measured concentrations of arsenic, our Fe@NCNT-CF nanostructures showed very high adsorption capacity and, when compared with our previously reported graphene-carbon nanotubes-iron (G-CNT-Fe) [42] or with magnetite decorated carbon fiber [43], the absorption capacity is almost double. This outstanding performance of arsenic absorption by flexible Fe@NCNT-CF hybrid nanostructures can be attributed to the high meso-porosity and open pore network, which facilitate the efficient and optimal capture of arsenic moieties by the well dispersed, highly concentrated, uniformly dispersed, and spatially separated iron oxide nano-particles. We carried out in-depth XPS analysis of arsenic adsorbed Fe@NCNT-CF in order to prove this point, and the survey scans from 0 to 250 eV (Figure 5b) show four prominent peaks at 43.7, 142.64, 206.88, and 231.62 eV, corresponding to As 3d, As 3p, As 3s, and As Auger, respectively. Besides this, the Fe 3s peak that was observed at 86 to 104 eV in Fe@NCNT-CF almost disappeared due to the anionic adsorption of arsenic on iron moieties which is also reflected in the deconvoluted high resolution As 3d spectra (Figure 5c), which shows, in addition to the usual two prominent peaks at 44.5, and 45.4 eV associated with As_2_O_5_ and As_2_O_3_, a weaker but visually discernible peak at 41.35 eV, which can be attributed to the binding of arsenic to ‘non-oxidized metal’, As-Fe-N in our case. These peaks are also reflected in the deconvoluted Fe 2p spectra of Fe@NCNT-CF and arsenic adsorbed Fe@NCNT-CF plotted in Figure 5d,e, respectively, which shows, besides the two prominent Fe 2p _3/2_ and Fe 2p _1/2_ peaks, an additional small peak at 719.86 eV attributed to As-Fe-N can be observed. These results prove that our newly developed Fe-NCNT@CF can effectively remove arsenic from contaminated water.

## 4. Conclusions

In summary, we report the microwave synthesis of iron and nitrogen co-doped carbon nanotubes (Fe@NCNT-rGO) that were obtained from renewable, sustainable, and relatively affordable naicin, Vitamin B_3_ precursors. Structural analysis by SEM and HRTEM revealed that Fe@NCNT-rGO possesses a well-defined millimeter long, bamboo shaped carbon nanotubes with nanometer sized iron nanoparticles that are embedded in protective shells along the tube walls. HRTEM elemental EDS maps show excellent dispersion and even distribution of iron and nitrogen moieties along the walls of nanotube, whereas XPS analysis showed that the nitrogen moieties are in the states of pyridine–N, pyrrole–N bands, and Fe–Nx–C, which all contribute to the superior ORR activity. The synergistic effect of the 3D mesoporous structure and composition results in excellent ORR catalytic activity that is comparable to that of traditional Pt/C catalyst. The excellent ORR catalytic efficiency of our microwave synthesized non-precious metal Fe@NCNT-rGO electro-catalysts makes it suitable for its application in fuel cells and metal–air batteries. The utility of Fe@NCNT-CF for the effective removal of arsenic from contaminated water at different pH values is also demonstrated.

## Supplementary Materials

The following are available online at https://www.mdpi.com/1996-1944/13/7/1686/s1, Figure S1: XPS Survey scan to quantify the chemical composition of vitamin derived Fe@NCNT-rGO materials.
