# Supplementary material for: Vitamin Derived Nitrogen Doped Carbon Nanotubes for Efficient Oxygen Reduction Reaction and Arsenic Removal from Contaminated Water
Source: Materials (Basel). 2020 Apr 4;13(7):1686. doi: 10.3390/ma13071686 (PMC7178648; doi:10.3390/ma13071686)
Supplement: Supplementary file 1 [file materials-13-01686-s001.pdf]

materials

Supplementary Materials

Vitamin Derived Nitrogen Doped Carbon Nanotubes for Efficient Oxygen Reduction Reaction and Arsenic Removal from Contaminated Water

Vadahanambi Sridhar [1], Kwang Hyo Jung [2], and Hyun Park [1,2,*]

1 Global Core Research Centre for Ships and Offshore Plants (GCRC-SOP), Pusan National University, Busan 46241, Republic of Korea; sridhar@pusan.ac.kr
2 Department of Naval Architecture and Ocean Engineering, Pusan National University, Busan 46241, Republic of Korea; kjung@pusan.ac.kr
* Correspondence: hyunpark@pusan.ac.kr; Tel.: +82-51-510-2730

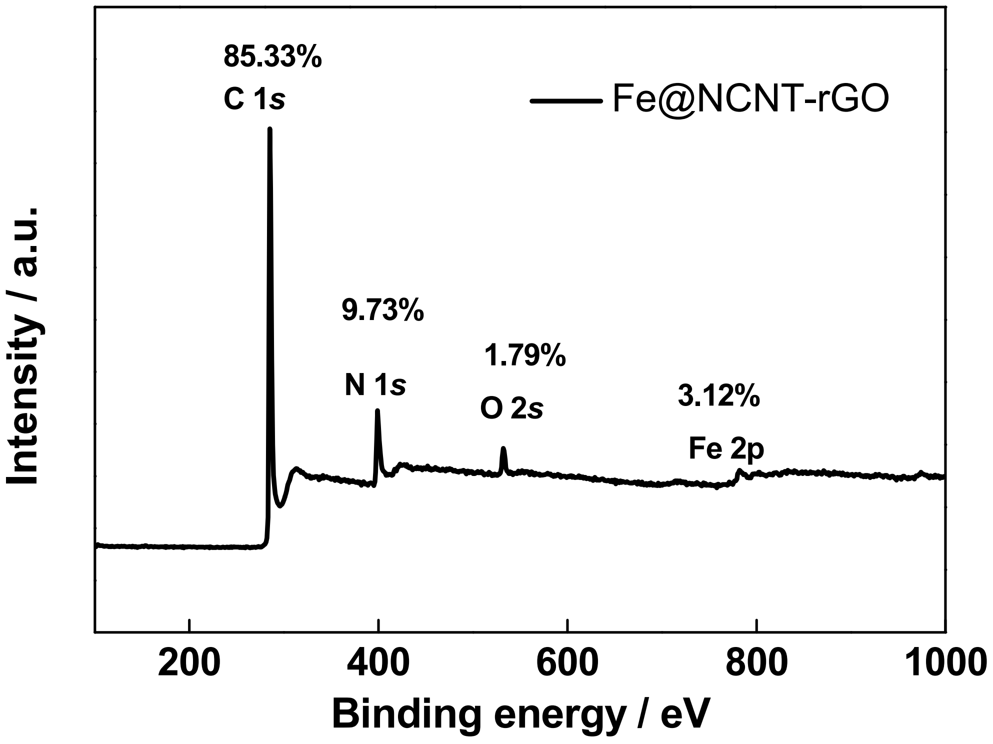

Figure S1. XPS Survey scan to quantify the chemical composition of vitamin derived Fe@NCNT-rGO materials.

© 2020 by the authors. Submitted for possible open access publication under the terms and conditions of the Creative Commons Attribution (CC BY) license (http://creativecommons.org/licenses/by/4.0/).
